# Early Life Stress Produces Compulsive-Like, but Not Impulsive, Behavior in Females

**DOI:** 10.1037/bne0000059

**Published:** 2015-06

**Authors:** Nichola M. Brydges, Megan C. Holmes, Anjanette P. Harris, Rudolf N. Cardinal, Jeremy Hall

**Affiliations:** 1 Centre for Cardiovascular Science, The University of Edinburgh; 2 Neuroscience and Mental Health Research Institute, Cardiff University School of Medicine; 3 Behavioral and Clinical Neuroscience Institute, Department of Psychiatry, University of Cambridge; 4 Liaison Psychiatry Service, Cambridgeshire; 5 Peterborough NHS Foundation Trust, Cambridge Biomedical Campus, United Kingdom; 6 Centre for Cardiovascular Science, The University of Edinburgh; 7 Neuroscience and Mental Health Research Institute, Cardiff University School of Medicine

**Keywords:** compulsivity, impulsivity, perseveration, childhood stress, sex differences

## Abstract

Adverse experiences during childhood are associated with the development of psychiatric disorders later in life. In particular, childhood abuse and neglect are risk factors for addictive disorders, such as substance misuse and pathological gambling. Impulsivity and compulsivity are key features of these disorders. Therefore, we investigated whether childhood adversity might increase vulnerability for addictive disorders through promotion of compulsive and impulsive behaviors. Rats were exposed to a brief, variable childhood or prepubertal stress protocol (Postnatal Days 25–27), and their behavior in a delay discounting task was compared with that of control animals in adulthood. Prepubertal stress produced compulsive-type behavior in females. Specifically, stressed females displayed inappropriate responses during a choice phase of the task, perseverating with nosepoke responding instead of choosing between 2 levers. Stressed females also showed learning impairments during task training. However, prepubertal stress was not associated with the development of impulsive behavior, as rates of delay discounting were not affected in either sex. Childhood adversity may contribute to the establishment and maintenance of addictive disorders by increasing perseveration in females. Perseverative behavior may therefore provide a viable therapeutic target for preventing the development of addictive disorders in individuals exposed to childhood adversity. These effects were not seen in males, highlighting sex differences in response to early life stress.

Exposure to adversity early in life is associated with the development of a range of psychiatric disorders later in life (Bale et al., 2010; Green et al., 2010; Kessler et al., 2010; Patchev, Rodrigues, Sousa, Spengler, & Almeida, 2014). Robust links exist between an adverse intrauterine environment and several chronic conditions in adulthood, and the “fetal origins of adulthood disease” hypothesis is empirically well supported (Braun, Challis, Newnham, & Sloboda, 2013; Schuurmans & Kurrasch, 2013; Wojcik, Lee, Colman, Hardy, & Hotopf, 2013). However, little is known about the long-term effects of stress experienced during the childhood or prepubertal phase of life. The prepubertal brain displays significant structural and functional differences to both the perinatal and adult brain. In particular, corticolimbic structures such as the hippocampus, amygdala, and prefrontal cortex (PFC) are maturing throughout childhood and adolescence (Brenhouse & Andersen, 2011; Gogtay et al., 2004; Pfefferbaum et al., 1994). Childhood adversity is associated with the development of several psychiatric disorders in adulthood (Bale et al., 2010; Green et al., 2010; Kessler et al., 2010; Lovallo, 2013; Sinha, 2008; Turecki, Ernst, Jollant, Labonté, & Mechawar, 2012), and consequently, the childhood phase is increasingly recognized as a sensitive period during which the brain may demonstrate specific vulnerabilities to the effects of stress (Eiland & Romeo, 2013; Pechtel & Pizzagalli, 2011).

Childhood adversity is a significant risk factor for substance misuse and other addictive disorders later in life (Andersen & Teicher, 2009; Lovallo, 2013). Impulsive and compulsive behavioral traits are associated with these disorders (Everitt & Robbins, 2013; Hosking & Winstanley, 2011; Koob & Volkow, 2010; Leeman & Potenza, 2012), but whether this relationship is causal or consequential is not clear (Dick et al., 2010). If causal, then exposure to adverse environmental factors, such as childhood adversity, may increase risk for addictive disorders through increasing impulsive and compulsive behavior. Impulsivity is thought to play a role in initiating addictive behaviors; for example, rats categorized as highly impulsive were more likely to acquire cocaine self-administration and at significantly faster rates than those classed as less impulsive (Dalley, Everitt, & Robbins, 2011; Perry, Nelson, & Carroll, 2008). One measure of impulsivity is delay discounting (a form of choice impulsivity), which refers to the decline in the perceived value of a reward as a function of increasing delay to receipt (Odum, 2011). Preference for a smaller, immediate reward over a larger but delayed reward is defined as an *impulsive choice*, whereas preference for a larger delayed reward is a *self-controlled choice* (Odum, 2011). On the other hand, *compulsivity*, defined as repetitive action inappropriate to the situation, is thought to promote maintenance of addictive behaviors (Leeman & Potenza, 2012; Morton & Munakata, 2002). *Perseveration* is a form of compulsive behavior, and is generally regarded as “a tendency to respond persistently to a particular stimulus, even after the response has become inappropriate or unrewarded” (Ersche et al., 2011, p. 754). Both delay discounting and perseveration are increased in substance misuse and gambling disorders (de Ruiter et al., 2009; Ersche et al., 2011; Volkow & Baler, 2014).

In the present study, we aimed to investigate whether childhood or prepubertal stress might increase risk for addictive disorders by increasing compulsive-type and impulsive behavior in adulthood. We used a delay discounting task and hypothesized that animals exposed to early life stress would exhibit increased delay discounting behavior (impulsivity) and higher levels of perseverative responding (compulsive-type behavior) when compared with control animals in adulthood. We tested male and female animals, as there is evidence for sex differences in the development of many psychiatric disorders (Bao & Swaab, 2010; Goel & Bale, 2009).

## Method

### Animals

Nineteen female and 33 male Lister hooded rats were bred in house from 11 adult pairs (Charles River, Tranent, UK). After weaning (Postnatal Day [PND] 21), animals were weighed weekly and housed in groups of two and three for the duration of the experiment in standard, same-sex, same-litter cages (61 cm × 43.5 cm × 21.5 cm high) lined with wood shavings (Lillico UK), on a 12:12 hour light–dark cycle with food (standard rat chow, RM1, Special Services Diet; Lillico, Surrey, UK) and water ad libitum. Temperature and humidity were maintained between 19 °C and 21 °C and 45% and 60%, respectively. Six litters were randomly assigned to the prepubertally stressed (PPS) group, the remaining five litters were used as controls (control group). In total, 9 females and 19 males composed the PPS group; 10 females and 14 males, the control group. Rats were identified by rings of permanent marker around the tail, and killed via a rising concentration of CO_2_ at the end of the experiment. All procedures were carried out in accordance with the UK Home Office Animals (Scientific Procedures) Act (1986) and local ethics guidelines.

### Prepubertal Stress

Animals were subjected to a brief, variable PPS protocol, which has been described previously (Brydges, Seckl, et al., 2014; Brydges, Wood, Holmes, & Hall, 2014). Briefly, on PND 25, animals experienced a 10-min swim stress in an opaque swim tank (25 cm high, 34 cm diameter, 12-L capacity) filled with 6 L of 25 ± 1 °C water. On PND 26, animals were placed into plastic restraint tubes (15 cm length, 5 cm diameter) for three sessions of 30 min, separated by 30-min breaks in the home cage. On PND27, animals were given 6 × 0.5mA, 0.5s foot shocks over 3 min (one every 30 s) in a rat operant box (30 cm × 25 cm, 32 cm high, 16 shock bars; Coulbourn Instruments, Lehigh, PA).

### Delay Discounting Task

Once animals had reached adulthood (PND 60), they were handled daily for 5 min and began gradual food restriction over one week. Animals were maintained between 85% and 90% of their free-feeding weight for the duration of the experiment. Experiments took place between 0800 and 1500 hours, and individual subjects were tested at a consistent time of day in the same operant chamber. Animals were given free access to food for 2 hr daily after testing.

### Apparatus

Four identical operant conditioning chambers were used (rat modular chamber, Campden Instruments, Loughborough, Leicestershire, UK). Inside each chamber there was an overhead house light, two retractable levers (left and right) and a food tray between the levers into which 45 mg sucrose reward pellets (Campden Instruments, Loughborough, Leicestershire, UK) could be delivered. The food tray had its own light, and an infrared beam allowing head entry into the food tray (nosepokes) to be recorded. The chambers were enclosed in sound-attenuating boxes. The Whisker control system (Cardinal & Aitken, 2010) was used to run a standard prepared schedule for training and the main delay discounting task (task phase). The training and task phase were based on previous reports (Cardinal & Howes, 2005), and are outlined as follows.

### Delay Discounting—Training Phase

Rats were initially trained to press levers (lever training). During a 30-min session, animals could press the left lever without limit, each press resulting in the immediate delivery of a single reward pellet. The lever was never retracted during this stage, and animals continued with daily sessions until they had obtained a cumulative total of 50 pellets. This was then repeated for the right lever. Rats were then moved onto nosepoke training—here they were trained to nosepoke to initiate presentation of a lever. Each trial began with levers retracted and the chamber in darkness. Every 40s, the houselight and traylights were illuminated, indicating the start of a trial. The subject had a maximum of 10 s to make a nosepoke response, or the trial was aborted and the chamber returned to darkness. If the subject nosepoked within 10 s the traylight was extinguished and a single lever presented. The rat had 10 s to respond on the lever, otherwise the lever was retracted and the chamber darkened. If the rat responded, a single pellet was delivered immediately and the traylight illuminated until the pellet was collected (or 10 s had elapsed, and the chamber was then darkened). In every pair of trials each lever was presented once (left and right), with the order of presentation random within each pair. Rats were trained to a criterion of 60 successful trials in 1 hr (maximum possible, 90 successful trials), in one training session per day. They were then moved onto the task phase.

### Delay Discounting—Task Phase

Animals were given daily sessions consisting of five delay blocks, with each delay block containing 12 trials. Sessions continued for 19 days, to ensure stable baseline behavior was achieved. Each daily session lasted 100 min, and each trial lasted 100 s, regardless of choice by subject. Trials began with levers retracted and lights out (intertrial state). Onset of the houselight signaled the start of the trial, the rat then had 10 s to nosepoke in the food tray to trigger presentation of a lever or levers. The first two trials of each delay block were forced-choice trials—only one lever was presented (one trial for each lever). If a rat failed to respond within 10 s with a nosepoke to the food tray or on a lever within 10 s of presentation (choice phase), an omission was scored and the box was returned to the intertrial state until the next trial was scheduled to begin. The remaining 10 trials within each delay were free-choice trials, and both levers were presented. Responding on one lever (designated Lever A) always resulted in the delivery of 1 pellet immediately; the other lever (designated Lever B), the delivery of 4 pellets after a varying delay (delay phase). Designation of left and right levers as A and B was counterbalanced between groups and sexes. As the delay blocks progressed, the delay to the larger (4 pellets—Lever B) reward was increased from 0 s in the first delay block to 10 s in the second delay block, 20 s in the third delay block, 40 s in the fourth delay block, and 60 s in the fifth delay block. Delay to the smaller (1 pellet—Lever A) reward was always 0 s. After the appropriate delay, the onset of the traylight signaled food delivery, after which the box was returned to the intertrial state (intertrial interval).

### Data Analysis

All data were analyzed using generalized linear models (JMP statistical software; SAS Institute, Cary, NC), and checked for normality of distribution and homogeneity of variance. Where these assumptions were not met, transformations were applied to produce closest approximations, and are noted in the results. Several transformations were tried for each nonnormal data set before further analysis, and the best transformation for the each data set was selected (the transformation that produced the best fit to normality and homogeneity of variance). Animal identity was nested within litter and group, and litter nested within group and these terms were added as random factors into all models to account for multiple measurements on the same animal and the use of multiple animals per litter (Myers, Well, & Lorch, 2010). Interactions between all terms in each model were also fitted. Post hoc Tukey’s honestly significant difference tests were used to further investigate significant results, and main and significant results are presented in the text. The method of food restriction was intended to produce similar weight reductions in both sexes, but actual mean weight loss was 15% in males and 10% in females. Therefore, percentage weight loss was also included in all analyses, but did not predict behavior (over and above sex). To assess task acquisition in the training phase, the effects of group and sex on the number of sessions taken to obtain 50 left and 50 right lever presses, and to complete 60 correct trials in one session during nosepoke training were analyzed. To assess learning during the task phase, the effect of session, group, sex, and delay block on number of responses for the large reward (Lever B) and total number of choices were analyzed. Responding had become stable (i.e., “day” was no longer a significant factor and stable baseline behavior was achieved) for both groups and sexes by Session 11, therefore in the following analyses, data from only Sessions 12 to 19 were used. To assess motivation and participation in the task phase, we set up models to analyze the effect of group, sex, and delay block on total number of trials initiated (nosepoke into food tray to initiate trial and presentation of levers) and total number of trials responded to (choosing a lever once presented). To assess response latencies, the effects of group, sex, and delay block on latency to initiate trials, respond to levers once presented, and collect the reward were analyzed. Perseveration was assessed through models investigating the effect of group, sex, and delay block on time spent nosepoking into the food tray during the choice phase, delay phase (divided by delay experienced), intertrial interval (divided by intertrial interval experienced), and reward collection phase. As numbers of trials responded to were quite low for some groups in later delay blocks, pairwise correlations were used to investigate the relationship between total number of responses and percentage of responses for the large reward (Lever B). At response rates of 0%–40%,there was a positive correlation between number of responses and proportion of those responses that were for the large reward. Therefore, only delay blocks with 40% responding and above were used in the following analysis: To assess choice impulsivity (delay discounting) the effects of sex, delay block, and group on proportion of responses for the large reward were assessed. However, it is worth noting that inclusion of trials with less than 40% responding did not alter the significance of results. A final model investigated the effects of group, sex, and age on body weight before adulthood testing began.

## Results

### Training Phase

#### Lever training—learning

**Figure 1 fig1:**
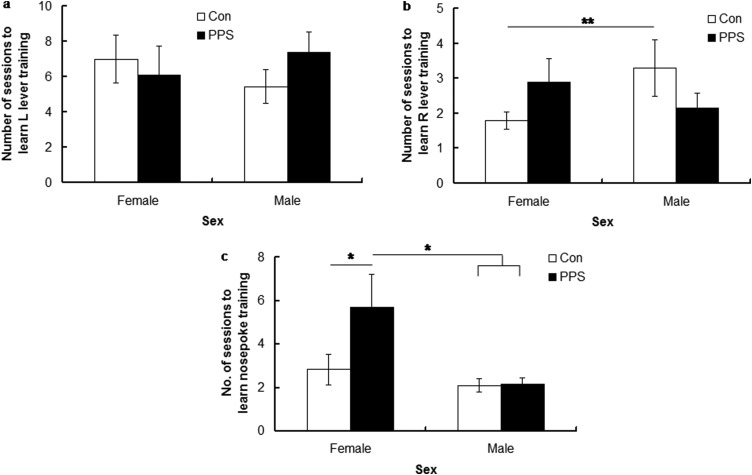
Number of sessions for control (Con) and prepubertally stressed (PPS) male and female rats to (a) learn to press the first (left) lever for a reward, (b) learn to press the second (right) lever for a reward, and (c) reach criterion in nosepoke training. Raw data are presented. Error bars represent one standard error. * *p* < .05. ** *p* < .01.

Figures 1a and 1b show the number of sessions taken for animals to acquire left and right lever training. Neither PPS nor sex affected learning to press the first (left) lever for a reward (Box–Cox transformed; Box & Cox, 1964); group, *F*(1, 5.65) = 0.0001, *p* = .99; sex, *F*(1, 35.26) = 0.005, *p* = .95; Group × Sex, *F*(1, 35.26) = 2.28, *p* = .14. However, control females took fewer sessions than control males to learn pressing the second (right) lever 50 times; log transformed, Group × Sex, *F*(1, 47.15) = 6.51, *p* = .01, although there were no main effects of group, *F*(1, 6.43) = 0.28, *p* = .61, or sex, *F*(1, 47.15) = 0.45, *p* = .51.[Fig-anchor fig1]

#### Nosepoke training—learning

Figure 1c illustrates that PPS females took longer than control females and all males to reach criterion in the nosepoke task; group, *F*(1, 25.1) = 4.7, *p* = .035; sex, *F*(1, 54.9) = 10.28, *p* = .002; Group × Sex, *F*(1, 23.94) = 4.48, *p* = .04.

### Task Phase

#### Learning

Number of lever presses for the large reward (Lever B) became stable (i.e., session was no longer a significant factor) by Session 6 in control females, Session 8 in PPS females, Session 4 in control males, and Session 3 in PPS males; arcsine transformed: Session × Group × Sex: *F*(18, 4507) = 2.16, *p* = .003. Overall responding became stable (i.e., session was no longer a significant factor) by Session 11 in both groups and sexes; arcsine transformed, day, *F*(18, 4507) = 2.12, *p* = .004.

#### Number of trials initiated

**Figure 2 fig2:**
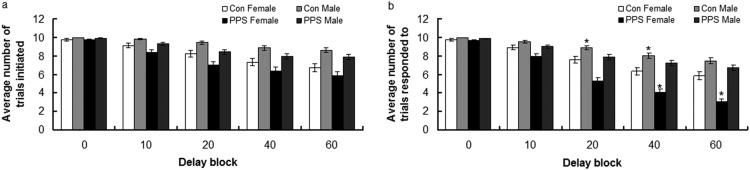
Average number of trials (a) initiated and (b) responded to by control (Con) and prepubertally stressed (PPS) male and female rats. Raw data are presented. Error bars represent one standard error. * *p* < .05.

Figure 2a illustrates that animals initiated fewer trials (through nosepoking into the food tray) as delay blocks progressed; arcsine transformed, delay block, *F*(4, 2012) = 119.23, *p* < .0001. Although the exact pattern of decreasing initiations differed between groups, it was not altered by PPS; group, *F* (1, 7.33) = 1.28, *p* = .29; sex, *F*(1, 46.91) = 0.003, *p* = .96; or Group × Sex interaction, *F* (1, 38.04) = 0.04, *p* = .85.[Fig-anchor fig2]

#### Number of trials responded to

Figure 2b shows that PPS females responded less to presented levers than all other groups during Delay Blocks 40 and 60, whereas control males responded more than all other groups during Delay Blocks 20 and 40; arcsine transformed, Group × Sex × Delay Block, *F*(4, 2012) = 3.66, *p* = .006. Stressed females responded less as delays increased, control females and stressed males responded less until delays of 40 seconds, with no difference at delays of 40 and 60 seconds, and control males responded more at delays of 0 and 10 seconds than all other delays, and less at delays of 40 and 60 than all other delays; Group × Sex × Delay, *F*(4, 2,012) = 3.66, *p* = .006.

#### Latencies

All animals initiated trials (beginning the trial by nosepoking into the food tray) more slowly in the final (60) compared with the first (0) delay block; log transformed, delay block, *F*(4, 3,674) = 29.44, *p* < .0001, but this was not affected by PPS; group, *F*(1, 0.28) = 0.18, *p* = .83; sex, *F*(1, 7.17) = 0.67, *p* = .44; or Group × Sex interaction, *F*(1, 9.9) = 1.01, *p* = .34).

**Figure 3 fig3:**
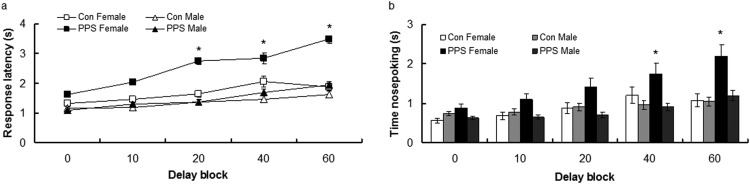
(a) Response latency and (b) time spent nosepoking during the choice phase for control (Con) and prepubertally stressed (PPS) male and female rats. Raw data are presented. Error bars represent one standard error. * *p* < .05.

Figure 3a illustrates response latency once levers were presented (after trial had been initiated). Response latency increased for all animals as delay blocks progressed, although the exact pattern differed between groups and sexes; log transformed, delay block, *F*(4, 1,892) = 88.33, *p* < .0001. PPS females had a longer response latency than PPS males in Delay Blocks 20 and 40, and than control females and all males in the final delay block (60); log transformed, Group × Delay Block × Sex, *F*(4, 1,892) = 2.44, *p* = .045. Overall, females had longer response latencies than males; sex, *F* (1, 41.69) = 4.59, *p* = .04.[Fig-anchor fig3]

Females had a longer latency to collect rewards than males in Delay Block 60, and females had a longer collection latency in Delay Block 60 than Delay Block 0; power transformed, Delay Block × Sex, *F*(4, 3,194) = 242, *p* = .046.

#### Nosepoking—compulsive behavior

Figures 3b shows the amount of time animals spent nosepoking during the choice phase (lever presentation). PPS females spent significantly longer than control females and all males nosepoking into the food tray during Delay Blocks 40 and 60; power transformed, Group × Delay Block × Sex, *F*(4, 1,876) = 2.57, *p* = .04. All animals increased time spent nosepoking as delay blocks progressed; delay block, *F*(4, 1,892) = 16.37, *p* < .0001. There was no main effect of PPS; group, *F*(1, 8.26) = 0.19, *p* = .67; sex, *F*(1, 41.33) = 3.84, *p* = .06; or Group × Sex interaction, *F* (1, 44.81) = 2.11, *p* = .15.

During the delay phase, animals spent less time nosepoking as delay blocks increased from 0 to 40, but there was no difference between Delay Blocks 40 and 60; delay block, *F*(3, 2,859) = 58.29, *p* < .0001. There was no effect of group, *F*(1,8.03) = 0.003, *p* = .96; sex, *F*(1, 45.73) = 3.04, *p* = .09; or Group × Sex interaction, *F*(1, 46.69) = 0.03, *p* = .87, on time spent nosepoking during the delay phase.

Females spent less time nosepoking during the intertrial interval (ITI) in Delay Blocks 40 and 60 compared with all other blocks, whereas males spent less time nosepoking in Delay Blocks 20, 40, and 60 than 0 and 10; log transformed, Delay Block × Sex, *F*(4, 3,676) = 3.4, *p* = .009. There were no differences between groups, *F*(1, 1.16) = 1.12, *p* = .46, or sexes, *F*(1, 19.88) = 0.34, *p* = .57, in amount of time spent nosepoking during the ITI. During reward collection, animals spent less time nosepoking as delay blocks progressed to from 0 to 20, but there was no difference between Delay Blocks 20, 40, and 60; delay block, *F*(4, 3,686) = 86.75, *p* < .0001. There was no effect of PPS; group, *F*(1, 47) = 0.02, *p* = .89; sex, *F*(1, 47.01) = 0.95, *p* = .34; or Group × Sex interaction, *F*(1, 47) = 0.15, *p* = .7.

#### Delay discounting—impulsive behavior

**Figure 4 fig4:**
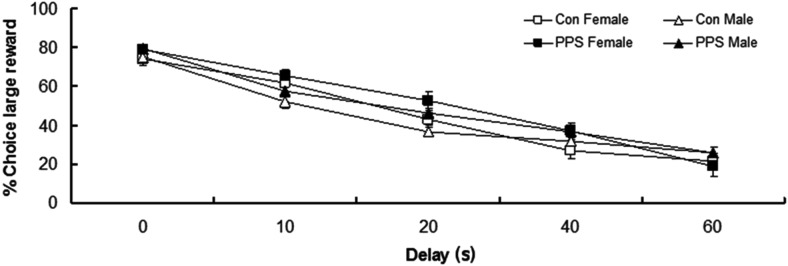
Percentage choice of large reward as delays to large reward increase for control (Con) and prepubertally stressed (PPS) male and female rats. Raw data are presented. Error bars represent one standard error.

Figure 4 shows percentage choice of large reward as the delay to the large reward increased. Males selected the large reward less as delays increased, whereas females selected the large reward less as delays increased to 40 seconds, but showed no difference between delays of 40 and 60 seconds; Sex × Delay block, *F*(4, 1,615) = 3.18, *p* = .004; see Figure 4. There were no main effects of PPS; group, *F*(1, 8.42) = 0.02, *p* = .88; sex, *F*(1, 42.18) = 3.68, *p* = .06; or Group × Sex interaction, *F*(1, 44.83) = 0.12, *p* = .73, on percentage choice of large reward.[Fig-anchor fig4]

#### Body weight PND 21–56

**Figure 5 fig5:**
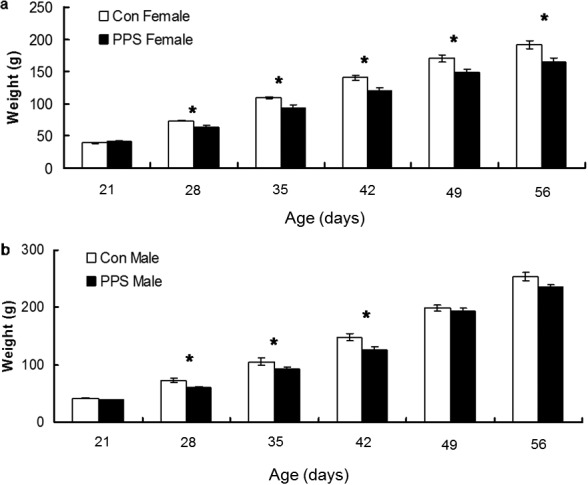
Weights of control (Con) and prepubertally stressed (PPS) (a) female and (b) male rats. Raw data are presented. Error bars represent one standard error. * *p* < .05.

Body weight (Box–Cox transformed) did not differ between groups the week before PPS was administered (PND 21); however, PPS resulted in reduced body weight in male and female animals by PND 28, and this lasted until PND 56 in females and PND 42 in males; Sex × Week × Group interaction, *F*(5, 228.1) = 2.59, *p* = .03; see Figure 5. Males were heavier than females from PND 49; Sex × Week × Group interaction, *F*(5, 228.1) = 259, *p* = .03; see Figure 5.[Fig-anchor fig5]

## Discussion

Exposure to PPS resulted in compulsive-type behavior in females (perseverative nosepoking), whereas males experiencing PPS did not differ from controls. PPS did not produce impulsive behavior in either sex, as all groups exhibited similar rates of delay discounting as delays to the large reward increased. Learning was impaired in PPS females during both task training and the main task, an effect not seen in PPS males. PPS animals of both sexes weighed significantly less than controls during PND 28–56 (females) and PND 28–42 (males), an effect previously reported in this model (Brydges, Hall, Nicolson, Holmes, & Hall, 2012; Brydges, Seckl, et al., 2014; Brydges, Wood, et al., 2014).

### Learning

Females experiencing PPS demonstrated learning impairments during task training, taking significantly longer than any other group to reach criterion during nosepoke training. This deficit was not observed in males experiencing PPS or in the preceding lever training phase. This indicates PPS females were only impaired when the associations between stimuli became more complex: Lever training required the animal simply to press a lever to obtain a reward pellet, whereas nosepoke training necessitated a nosepoke response (in response to house and tray light illumination) followed by a lever press. PPS females also took longer than any other group to stabilize their proportion of reward selection (large vs. small) in the main task, again suggesting impairments in learning more complex associations. Neural processes mediating simple versus more complex components of appetitive operant conditioning are genetically dissociable, although neuroanatomical substrates underlying each stage are not well characterized (Malkki et al., 2010). However, there is evidence that the striatum underlies operant conditioning in general (Liljeholm & O’Doherty, 2012; Yin, Ostlund, Knowlton, & Balleine, 2005). Previous studies have shown PPS impairs learning under stress in two-way shuttle avoidance, a task which also depends on the striatum (Tsoory & Richter-Levin, 2006; Wietzikoski et al., 2012). In combination with the present study, this suggests that exposure to PPS may impair striatal function, resulting in impaired learning. However, further experiments are required to investigate this hypothesis further. Of relevance to addictive disorders, the dorsal striatum has been implicated in the development of compulsive drug seeking behavior, due to its role in stimulus-response habit learning (Everitt & Robbins, 2013). We have previously found that both male and female rats experiencing PPS display enhanced anxiety-type behavior (Brydges et al., 2012); however, we do not believe that enhanced anxiety was responsible for the learning impairments seen in stressed females: PPS females were able to learn the initial training task as well as other groups (simple lever press to obtain a food reward from the reward tray), and this impairment was not seen in males. Animals were moved on the main task only when they had reached the required criterion in the learning phase, so any initial delays in learning should not impact performance in later stages of the task.

### Compulsive Behavior

Exposure to PPS resulted in compulsive-type behavior in females: They made fewer responses to levers and instead spent more time nosepoking into the food tray during Delay Blocks 40 and 60. Here, PPS females appear to perseverate with a behavior that is no longer appropriate for the situation (nosepoking into the food tray instead of selecting a lever) as delays to a large reward increase. Perseveration tends to increase as demands on working memory increase, which may explain why this effect is only seen in Delay Blocks 40 and 60 (Stedron, Sahni, & Munakata, 2005). Compulsive behaviors (including perseveration) and impulsive behaviors are observed in a number of psychiatric conditions, including substance misuse and gambling disorders (Álvarez-Moya et al., 2009; Andersen & Teicher, 2009; de Ruiter et al., 2009; Lovallo, 2013). Whether compulsive and impulsive behaviors are a cause or a consequence of these disorders is a matter of some debate. A recent study found a positive correlation between number of childhood adversities and perseverative errors on the Wisconsin Card Sorting Test in a healthy population (mediated by catechol-O-methyltransferase genotype; Goldberg et al., 2013). Combined with the present study, this suggests that childhood adversity may contribute to the development of addictive disorders through increasing compulsive behavior later in life. It is interesting that maternal separation stress (at PND 9) results in perseverative behavior in both male and female rodents, whereas prenatal stress does not (Fabricius, Wörtwein, & Pakkenberg, 2008; Wilson, Schade, & Terry, 2012), suggesting that the timing and the nature of adversity are crucial for determining adulthood outcomes.

All animals initiated less and responded more slowly to fewer trials as delay blocks progressed, which likely reflects increasing satiety as rewards are earned. However, there were no differences between groups and sexes in the average number of trials initiated, suggesting that all groups were equally motivated to participate in the task as delay blocks progressed. It would be interesting to reverse the delays in this experiment and determine whether the same patterns of responding and perseveration are observed, particularly as recent studies have found that the manner in which delays are varied can alter delay discounting behavior (specifically the choice for the large reinforce; Maguire, Henson, & France, 2014; Tanno, Maguire, Henson, & France, 2014).

### Impulsive Behavior

We find no evidence that PPS increases choice impulsivity, as measured in a delay discounting task. In contrast, maternal separation stress during PND 2–21 in rodents resulted in decreased choice impulsivity (Lovic, Keen, Fletcher, & Fleming, 2011), again suggesting an important role for timing and type of adversity exposure. Studies in humans have produced mixed results, with some finding increased rates of delay discounting, others finding no change after exposure to childhood adversity (Acheson, Vincent, Sorocco, & Lovallo, 2011; Lovallo et al., 2013). A possible explanation for this discrepancy is that the exact timing of childhood adversity is not taken into account (e.g., early vs. late childhood). There is also evidence that genotype (e.g., D4 dopamine receptor variants) can interact with childhood adversity in determining choice impulsivity (Sweitzer et al., 2013). Impulsivity is a multifaceted construct, and exposure to prenatal stress and PPS in rodents, and childhood adversity in humans, is correlated with increased motoric impulsivity, which may increase risk for the initiation and maintenance of addictive disorders (Brydges et al., 2012; Lovic et al., 2011; Steiger et al., 2012). As impulsivity is such a multifaceted construct, another possible explanation for discrepancies in the literature is the way in which impulsivity is measured, for example, choice versus motoric impulsivity. On average, animals select the large reward 80% of the time in Delay Block 0 (no delay to the large or small reward). This level of selection is often seen in the first block in delay discounting studies with rats (Winstanley, Dalley, Theobald, & Robbins, 2003). We suggest that animals would not select this lever 100% of the time as they are likely to be sampling the other lever.

### Sex Differences

Exposure to PPS resulted in perseverative responding in female but not male animals, supporting the hypothesis that there are sex differences in the development of neuropsychiatric disorders after exposure to early life stress. This further highlights the need to consider males and females separately in preclinical models of neuropsychiatric disorders (Cahill, 2006; Simpson, Ryan, Curley, Mulcaire, & Kelly, 2012).

Control females learnt faster than control males during lever training, whereas males obtained stable numbers of presses for the large reward faster than females. Previous studies have sometimes found a female, sometimes a male, advantage in various tasks of learning and memory, and underlying mechanisms are thought to include differences in sex and stress hormones, neurogenesis-related processes, neurotrophic factors (e.g., brain-derived neurotrophic factor) and differences in the density of a variety of receptors in the brain, including dopamine and nerve growth factor receptors (Simpson & Kelly, 2012; Simpson et al., 2012). However, is not clear why these differences exist in the present study.

## Conclusion

Exposure to a brief, variable PPS protocol resulted in increased perseveration and impaired learning in adult female rats. This suggests that childhood adversity may contribute to the development and maintenance of addictive disorders through increasing compulsive-type behavior in females. Further studies are required to fully elucidate the mechanisms governing these alterations, and provide targets for therapeutic intervention.
